# *Streptococcus mutans* suppresses filamentous growth of *Candida albicans* through secreting mutanocyclin, an unacylated tetramic acid

**DOI:** 10.1080/21505594.2022.2046952

**Published:** 2022-03-21

**Authors:** Li Tao, Min Wang, Guobo Guan, Yuwei Zhang, Tingting Hao, Chao Li, Shuaihu Li, Yihua Chen, Guanghua Huang

**Affiliations:** a Department of Infectious Diseases, Huashan Hospital, Fudan University, Shanghai, China; b State Key Laboratory of Genetic Engineering, School of Life Sciences, Fudan University, Shanghai, China; c State Key Laboratory of Microbial Resources, Institute of Microbiology, Chinese Academy of Sciences, Beijing, China; d University of Chinese Academy of Sciences, Beijing, China; e State Key Laboratory of Mycology, Institute of Microbiology, Chinese Academy of Sciences, Beijing, China; f Shanghai Engineering Research Center of Industrial Microorganisms

**Keywords:** *Candida albicans*, *Streptococcus mutans*, mutanocyclin, filamentous growth, transcriptional regulation

## Abstract

Fungi and bacteria often co-exist and physically or chemically interact with each other in their natural niches. This inter-kingdom species interaction is exemplified by the gram-positive bacterial pathogen *Streptococcus mutans* and opportunistic fungal pathogen *Candida albicans*, which co-exist in the human mouth. It has been demonstrated that the dynamic interaction between these two species plays a critical role in their virulence and biofilm development. In this study, we discovered that *S. mutans* represses filamentous development and virulence in *C. albicans* through secreting a secondary metabolite, mutanocyclin (a tetramic acid). Mutanocyclin functions by regulating the PKA catabolic subunit Tpk2 and its preferential binding target Sfl1. Inactivation of Tpk2 in *C. albicans* results in an increased sensitivity to mutanocyclin, whereas overexpression of Tpk2 leads to an increased resistance. Dysfunction of *SFL1* and its downstream target genes overrides the hyphal growth defect caused by mutanocyclin. Further investigation demonstrates that three glycosylphosphatidylinositol (GPI)-anchored proteins (Spr1, Hyr4, and Iff8), associated with cell wall biogenesis and remodeling, and a set of filamentous regulators also contribute to the mutanocyclin response. We propose that both transcriptional regulation and cell wall composition contribute to mutanocyclin-mediated filamentous inhibition. This repressive effect of mutanocyclin could function as a natural regulator of filamentous development in *C. albicans*.

## Introduction

Interactions between bacteria and fungi are common in the environment and human body. These interactions include various modes ranging from mutualism to antagonism and may result in changes to the pathogenicity of one or both partners [1]. *Candida albicans* is a major opportunistic fungal pathogen of humans and exists as a commensal in the mouth, gastrointestinal tract (GI) tract, genital tract, and on the skin [2–4]. In these natural niches, many bacterial species co-exist with the fungus and affect its growth and pathogenicity [5,6]. For example, *Streptococcus mutans*, a major bacterial pathogen of dental caries, and *C. albicans* are common residents of the human oral cavity [7]. Both species have been thought to be tightly associated with the development of caries, especially in immunocompromised individuals [8–10]. Therefore, the inter-kingdom interaction between *S. mutans* and *C. albicans* has been considered to have a synergistic effect on this disease.

The *S. mutans-C. albicans* association has pleiotropic effects that could be antagonistic or cooperative. For example, the two species can form a dual biofilm that may enhance the virulence of *S. mutans* and the onset and severity of dental caries [11]. Within oral biofilms, the two species produce biofilm-derived metabolites that modulate gene expression and affect the cell behaviors and colony formation of each other [11,12]. *C. albicans* has been shown to induce expression of the *S. mutans* virulence factor glucosyltransferase B (GtfB). In turn, GtfB catalyzes the production of α-glucan and binds to the mannan layer of the *C. albicans* cell wall [13,14]. The cell wall-associated polypeptide of *S. mutans* antigen I/II mediates adhesion to *C. albicans* cells and promotes acid production within the dual-species biofilm [15]. Conversely, the relationship between *C. albicans* and *S. mutans* can be antagonistic. *S. mutans* produces quorum-sensing molecules (for example, CSP) or peptides (for example, SM-F2), which repress hyphal formation and virulence of *C. albicans* [16,17]. Therefore, the inter-kingdom species interactions among bacteria and fungi are complex. The interactions between *C. albicans* and *S. mutans* could be associated with a healthy and balanced community.

The ability of *C. albicans* to switch between the yeast and filamentous forms is critical to its virulence [4,18,19]. Filamentous cells are more invasive than yeast-form cells and cause extensive damage to host organs. Many host-associated environmental factors and multiple signaling pathways are involved in the regulation of yeast-filamentous transitions in *C. albicans* [4,18,19]. For example, high environmental temperatures, neutral ambient pH, high levels of CO_2_, and serum induce the development of filaments, whereas an acidic pH, low temperatures, and rich nutrient conditions are conducive to the yeast form of *C. albicans*. A recent report has shown that saliva facilitates the growth of *C. albicans* hyphae under in vitro culture conditions [20]. Among the regulators of morphological changes, the conserved cAMP/protein kinase A (PKA) pathway plays a major role in *C. albicans* [21,22]. Moreover, the Ste11-Hst7-Cek1/Cek2-mediated MAPK pathway, Rim101-mediated pH-sensing pathway, and Tup1/Nrg1 transcriptional repressors also play critical roles in the filamentous growth of *C. albicans* [23–27].

In this study, we report that *S. mutans* inhibits the development of filaments and affects the virulence of *C. albicans* by secreting a tetramic acid, mutanocyclin. This inhibitory effect of mutanocyclin could directly or indirectly affect the activity of the cAMP/PKA signaling pathway. We also found that a set of transcription factors, such as Efg1, Ahr1, Nrg1, Fcr1, and Sfl1, and several GPI-anchored proteins (Spr1, Hyr4, and Iff8) were involved in this regulation. Animal experiments demonstrated a protective effect of mutanocyclin on infections caused by *C. albicans*. Our findings uncover a novel link between the two species and provide new insights into the interspecies interactions between bacteria and fungi in the host.

## Materials and methods

### Strains, libraries, media, and culture conditions

C.*albicans* cells from stocks stored at −80°C were streaked on YPD agar plates (2% peptone, 1% yeast extract, 2% glucose and 2% agar, wt/vol) and incubated overnight at 30°C. Peptone and yeast extract were purchased from Oxoid Ltd. Company (Hants, UK). Glucose was from Beijing Chemical Works (Beijing, China) and agar was from Sangon Biotech (Shanghai, China). For routine culture, *C. albicans* cells were plated on YPD agar medium and incubated for 24 hours at 30°C. Cells of single colonies were then collected, dilutedand used for morphological analysis. *S. mutans* strains were cultured in brain heart infusion medium (BHI; Oxoid, Basingstoke, United Kingdom) plus .5% sucrose [28], referred to herein as BHI-sucrose medium [29]. Sucrose was purchased from Beijing Chemical Works (Beijing, China). BHI-sucrose and BHI + Lee’s glucose (50% Lee’s glucose mixed with 50% BHI broth) media were used for the filamentation assays [29,30]. To obtain supernatant, *S. mutans* was grown in liquid BHI-sucrose broth at 30°C. After 24 hours of incubation, the bacterial cultures were centrifuged, filtered, and stored at 4°C for further use. A deletion mutant library of 165 transcription factors and a mutant library of cell wall-related genes were used to screen essential elements involved in filamentation during interspecies interactions [31,32]. For library screening, *C. albicans* strain SN250 served as the WT control. All strains used in this study are listed in **Table S1**.

### Disruption of the mutanocyclin synthesis gene cluster in *S.* *mutans*

An in-frame deletion mutant of the *S. mutans* mutanocyclin gene cluster was constructed by two rounds of homologous recombination. The 2.1-kb counter selection marker IFDC2 cassette was amplified using the primer pair ldhF1-*Bsa*I/ermR1-*Bsa*I with pIFDC2 as a template [33]. A .7-kb and .8-kb fragment flanking the gene cluster were PCR amplified using primers 35C2-upF/35C2-upR-BsaI and 35C2-dnF-BsaI/35C2-dnR using the genomic DNA of *S. mutans* 35, a clinically isolated *S. mutans* strain, as a template [34]. The two fragments and IFDC2 cassette were ligated using the Golden Gate cloning assay and transformed into strain *S. mutans* 35. Colonies with resistance to erythromycin on BHI plates were selected and PCR-verified with primers 35C2-upF1/35C2-dnR1. The IFDC2 cassette was then removed. The .7-kb fragments flanking the IFDC2 cassette were PCR-amplified with primers 35C2-upF/35C2-upR3-BsaI and 35C2-dnF3-BsaI/35C2-dnR, ligated using Golden Gate cloning, and transformed to remove the IFDC2 cassette. Colonies resistant to *p*-Cl-Phe on BHI plates were selected and PCR-verified.

### Spectroscopic analysis

Detection of mutanocyclin in the pure culture of *S. mutans* and *S. mutans* Δ*muc* or in the mixed culture with *C. albicans* was performed using high-performance liquid chromatography (HPLC) with the chemically synthesized standard as a positive control. Briefly, the cells were cultured in BHI-sucrose medium at 30°C for 72 hours and subjected to centrifugation. The supernatants were extracted three times with an equal volume of ethyl acetate, evaporated under a vacuum, and dissolved in methanol. HPLC detection was carried out on a Shimadzu HPLC system (Shimadzu, Kyoto, Japan) using a C18 column (4.6 × 250 mm, 5 μm, Apollo, Alltech, Lexington, Kentucky, USA) under gradient elution conditions. The detection wavelength was 235 nm. The column was developed with solvent A (H_2_O with .1% (v/v) formic acid) and acetonitrile at a flow rate of 1.0 mL/min. The percentage of acetonitrile was maintained at 5% over 0–5 min, changed from 5 to 40% over 5–25 min and changed from 40 to 100% over 25–40 min.

### Chemical synthesis of mutanocyclin

Mutanocyclin was chemically synthesized from Boc-protected *D*-Leu as previously described [34]. Briefly, Boc-*D*-Leu was coupled with Meldrum’s acid in the presence of 1-(3-dimethylaminopropyl)-3-ethylcarbodiimide (EDC) hydrochloride and dimethylaminopyridine (DMAP). Cyclization of the lactam ring was achieved by heating in MeOH to provide *N*-protected tetramic acid. After removing the Boc group by TFA, C3-acetylation to generate mutanocyclin was performed with acetyl chloride (AcCl) in the presence of boron trifluoride-diethyl ether complex (BF3 OEt2).

### Growth curve assays

C. *albicans* cells of the WT strain SC5314 were initially grown in liquid YPD at 30°C for 24 hours and then collected and washed twice with 1x PBS (10 mM phosphate buffer, 2.7 mM KCl, 137 mM NaCl, pH 7.4). Approximately 1 × 10^5^ cells were inoculated into 3 mL of YPD medium plus DMSO containing 8 µg/mL, 16 µg/mL, or 32 µg/mL of mutanocyclin. The cells were incubated at 30°C with shaking at 200 rpm. Cell densities were detected at different time points by measuring the optical density at 600 nm (OD600).

### Filamentation assays

C. *albicans* cells were plated on YPD agar medium and incubated at 30°C for 24 hours. Approximately 1 × 10^4^ cells were inoculated intofilamentous growth medium containing *S. mutans* cells, the supernatant, or mutanocyclin. The samples were incubated at 30°C for 24 to 48 hours. The degree of filamentation was defined according to both the length and percentage of filamentous cells. Approximately 200 cells were counted in each sample.

### Subcellular localization of mutanocyclin

C. *albicans* cells (1 × 10^4^ cells/well) were incubated in liquid Lee’s glucose medium containing 32 µg/mL mutanocyclin or an equal volume of vehicle (.32% DMSO) at 30°C for 30 min or 24 hours. Cells were then collected and washed with 1x PBS. Images were obtained using a confocal laser scanning microscope. The excitation wavelength for mutanocyclin was 254 nm.

### Animals

All animal experiments were performed according to the guidelines approved by the Animal Care and Use Committee of Fudan University, approval code: SYXK(hu)2020–0032. Four-five weeks old female BALB/c mice weighing 18–20 g were used for animal experiments.

### Histopathological assay

Ex vivo tongue infection assays were performed as described previously [35]. The tongues were excised from sacrificed female BALB/c mice, and placed in each well of a 24-well plate. *C. albicans* cells (1 × 10^7^) in 5 μL of PBS containing DMSO (v/v, .32%) or mutanocyclin (32 μg/mL) were spotted on the tongues (ex vivo infection). After 24 hours of incubation at 37°C tongues were fixed in 10% buffered formalin, washed twice with PBS, dehydrated, and embedded in paraffin wax. Paraffin blocks were sectioned and stained with periodic acid-Schiff (PAS) for microscopy assays [36].

### Scanning electron microscopy (SEM) assay

SEM assays were performed as described previously with slight modifications [35]. *C. albicans* cells (1 × 10^7^) in 5 μL of PBS containing DMSO (v/v, .32%) or mutanocyclin (32 μg/mL) were spotted on the tongues excised from sacrificed female BALB/c mice (ex vivo infection). After 24 hours of incubation at 37°C tongues were fixed in 2.5% glutaraldehyde, and washed three times with PBS. The samples were then dehydrated using gradually increasing concentrations of ethanol, dried, and coated with gold. The prepared samples were imaged using a Phenom Prox Scanning Electron Microscope.

### *Galleria mellonella* infection model

To determine the in vivo effects of mutanocyclin, a *G. mellonella* survival assay was performed according to a previously described methodology [37]. *G. mellonella* in the final stage of the larval phase and weighing approximately 300 mg were stored in the dark and used within 7 days of shipment. Ten microliters of *C. albicans* (SC5314) inoculums (1 x 10^8^ CFU/mL) containing only DMSO (v/v, .32%) or different concentrations of mutanocyclin was injected into larvae through a 10-µL microsyringe. A group of 20 larvae was used per concentration, and the larvae inoculated only with 1x PBS were used to show that death was not due to needle trauma. After injection, the larvae were incubated at 30°C and the number of dead *G. mellonella* was recorded at 12, 24, 36, 48, 60, and 72 hours. A larva was considered dead when it showed no response to touch.

### RNA extraction and quantitative real-time PCR (Q-RT-PCR)

Q-RT-PCR assays were performed according to our previous publications with modifications [30]. *C. albicans* cells were cultured in liquid Lee’s glucose medium as described in the main text. Cells were harvested, and total RNA was extracted using Thermo Scientific GeneJET RNA Purification kits (Waltham, USA) according to the manufacturer’s instructions. cDNA was synthesized with an oligo(dT) 18 primer (Tm, 32.1; 100 μM) from .6 µg of total RNA using Thermo Scientific RevertAid H Minus Reverse Transcriptase (Waltham, USA). Q-RT-PCR was performed using TOYOBO SYBR green master mix (QPS-201) on a Bio-Rad CFX96 real-time PCR detection system. The signal from each experimental sample was normalized to the expression of the *ACT1* gene.

### RNA-Seq analysis

C.*albicans* cells were spread on YPD agar plates and incubated at 30°C for 24 hours, after which they were inoculated into liquid Lee’s glucose medium containing DMSO (v/v, .32%) or mutanocyclin (32 µg/mL) and incubated at 30°C for 24 hours. Two biological repeats were performed for each condition. Cells were harvested, and total RNA was extracted as described above. RNA-Seq analysis was performed by the company Berry Genomics (Beijing, China) as described previously [30]. Briefly, approximately 10 million (M) reads were sequenced in each library of samples. The library products were sequenced using an Illumina NovaSeq platform. The raw reads were filtered by removing adapter and low-quality reads (Phred score ≤10) using the FASTX-Toolkit v.0.14. Clean reads were mapped to the genome of *C. albicans* SC5314 using HiSat v2.0.5 with default parameters. Mismatches less than two bases were allowed in the alignments. Relative gene expression levels were calculated using the Fragments Per kb per Million reads (FPKM) method, FPKM = 10^6^C/(NL/10^3^), where C is the number of fragments uniquely aligned to gene A, N represents the total number of fragments uniquely aligned to all genes, and L is the number of gene A bases. To be considered significantly differentially expressed, a gene must satisfy three criteria: (1) an FPKM value ≥20 at least in one sample, (2) a fold change value ≥1.5 (except for the functional categories sheet of Data set S1, in which a 2-fold change cutoff was used), and (3) an adjusted P-value (false discovery rate [FDR]) < .05. GO functional enrichment analysis was carried out according to GO terminology determined using the online CGD GO Term Finder tool (http://www.candidagenome.org/cgi-bin/GO/goTermFinder).

## Results

### S. mutans *inhibits filamentous growth of* C. albicans

**Figure 1.**
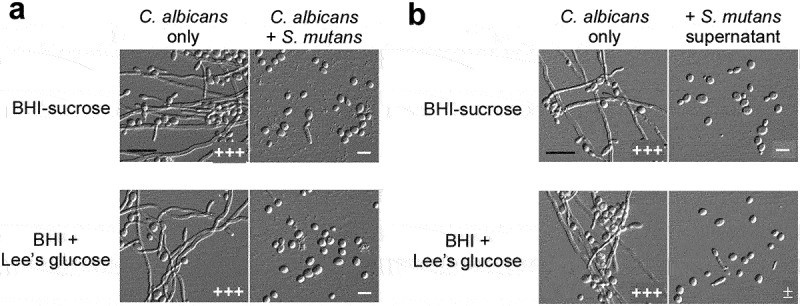
***S. mutans* inhibits filamentous growth of *C. albicans* at 30°C** . (a) *C. albicans* SC5314 cells (1 × 10^6^) were mixed with *S. mutans* cells (1 × 10^7^) and co-cultured in BHI-sucrose medium or BHI + Lee’s glucose medium at 30°C for 24 hours. (b) the supernatant of *S. mutans* was mixed with *C. albicans* SC5314 cells (1 × 10^6^) and incubated in BHI-sucrose medium or BHI + Lee’s glucose medium at 30°C for 24 hours. the “-” sign indicates that no filamentous cells were observed; “±”, “+”, “++”, and “+++”, represent 1–10%, 10–30%, 30–50%, and 50–70% of filamentous cells, respectively. Scale bar, 20 µm.

When grown in BHI-sucrose or BHI + Lee’s glucose medium at 30°C *C. albicans* was able to develop robust filaments (Figure 1 and S1). These culture conditions also support the growth of *S. mutans*. To explore the effect of *S. mutans* on the growth of filaments, we mixed *C. albicans* and *S. mutans* cells and cultured them statically in the same medium at 30°C and 37°C As shown in Figure 1a and S1a, the growth of *C. albicans* filaments was almost blocked in both media in the presence of *S. mutans* cells, revealing a remarkable inhibitory effect of the bacterial cells on the development of *C. albicans* filaments.

We suspected that *S. mutans* cells secreted some secondary metabolites into the medium that led to the inhibitory effect on *C. albicans* filamentation. Therefore, we next examined whether the *S. mutans* culture supernatant had a similar effect. As expected, the supernatant suppressed the development of filaments of *C. albicans* in both media (Figure 1b and S1b). Our findings indicate that *S. mutans* cells inhibit the growth of *C. albicans* filaments through secreting some unidentified chemicals.

### *Mutanocyclin, a tetramic acid, secreted by* S. mutans *cells inhibits* C. albicans *filamentation*

**Figure 2.**
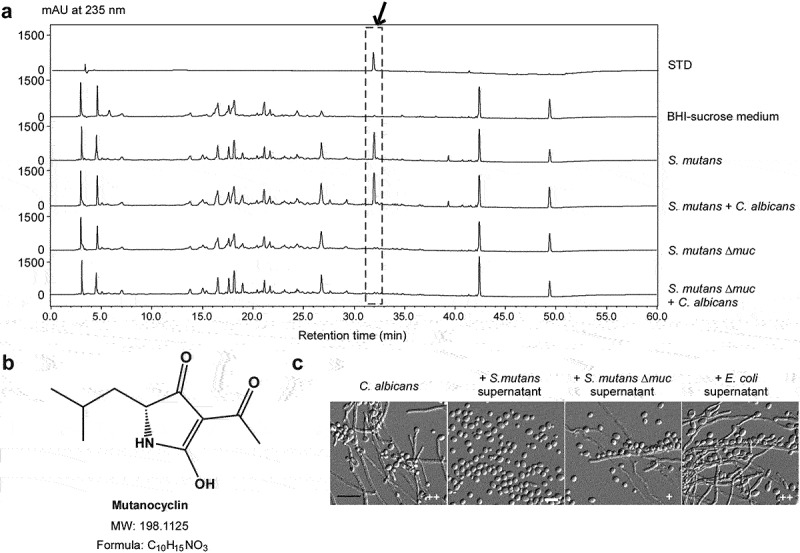
**Identification of a tetramic acid compound, mutanocyclin, produced by *S. mutans*, as an inhibitor of *C. albicans* filamentous growth**. (a) HPLC analysis of the supernatant extracts of the *S. mutans*, *S. mutans* mixed with *C. albicans*, *S. mutans* Δ*muc* mutant, and *S. mutans* Δ*muc* mixed with *C. albicans* cultures. The BHI-sucrose medium served as a negative control. The *X*-axis represents the retention time in minutes, and the *Y*-axis is the absorbance unit (mAU) at 235 nm. Chemically synthesized mutanocyclin served as the reference standard (STD). the black arrow indicates the peak of mutanocyclin. (b) Chemical structure of mutanocyclin. MW: molecular weight. (c) Morphologies of *C. albicans* (SC5314) in the presence of supernatant from *S. mutans*, *S. mutans* Δ*muc* mutant, or *E. coli* cultures. *C. albicans* cells (1 × 10^6^) were incubated in BHI + Lee’s glucose medium with or without an equal volume of the bacterial supernatant extracts at 30°C for 48 hours. the number of “+” signs indicates the degree of filamentation. the “-” sign indicates that no filamentous cells were observed; “+” and “++” represent 10–30% and 30–50% of filamentous cells, respectively. Scale bar, 20 µm.

In a previous study, we identified mutanocyclin as a rich secondary metabolite with anti-infiltration activity in *S. mutans* [34]. The mutanocyclin-encoding cluster is confined to approximately 15% *S. mutans* strains (including the *S. mutans* 35 used in this study) and some closely related species [38]. This chemical is produced under conditions mimicking host niches (for example, anaerobic conditions). We therefore performed high-performance liquid chromatography (HPLC) analysis and examined the production of mutanocyclin in *S. mutans* and *S. mutans-C. albicans* mixed cultures. As shown in Figure 2a, a peak with a retention time equivalent to that of chemically synthesized mutanocyclin was observed. High resolution-mass spectrum analysis of this compound from the *S. mutans* and *S. mutans-C. albicans* mixed cultures revealed a chemical formula of C_10_H_15_NO_3_ (calculated molecular weight: 198.1125), which was identical to mutanocyclin [34] (Figure 2b).

Since the biosynthetic gene cluster (*muc*) is essential for the production of mutanocyclin in *S. mutans*, we generated a Δ*muc* mutant in *S. mutans* 35 and tested the effect of this mutant on the growth of filaments in *C. albicans* in a co-culture system. As shown in Figure 2c, the Δ*muc* mutant of *S. mutans* was unable to produce mutanocyclin and allowed the growth of filaments in *C. albicans*. These results suggest that mutanocyclin produced by *S. mutans* cells has a direct repressive effect on *C. albicans* filamentation.

### *Mutanocyclin inhibits* C. albicans *filamentation in a dose-dependent manner*

**Figure 3.**
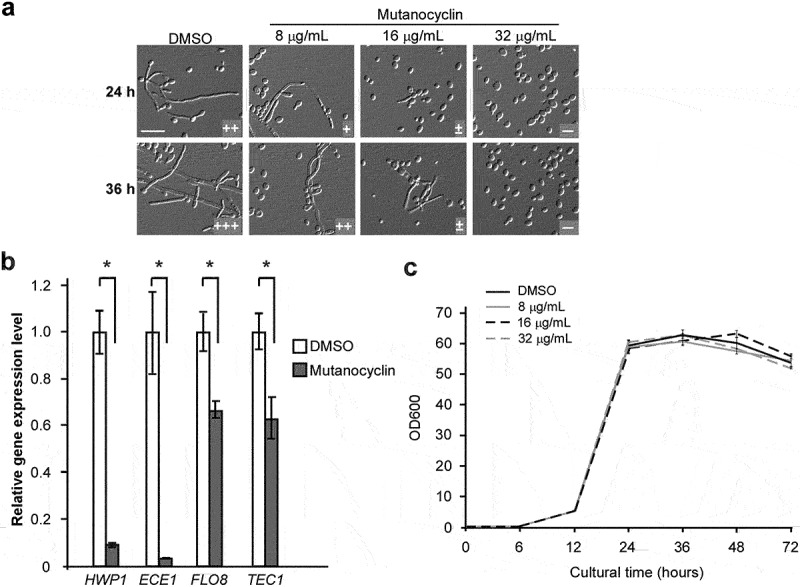
**Inhibitory effect of mutanocyclin on*C. albicans* filamentation**. (a) Morphologies of *C. albicans* cells in the presence of DMSO or 8, 16, and 32 µg/ml of mutanocyclin. *C. albicans* cells (SC5314, 1 × 10^4^) were incubated in BHI-sucrose medium with or without mutanocyclin at 30°C for 24 to 36 hours. the number of “+” signs indicates the degree of filamentation. the “-” sign indicates that no filamentous cells were observed; “±”, “+”, “++”, and “+++”, represent 1–10%, 10–30%, 30–50%, and 50–70% of filamentous cells, respectively. Scale bar, 20 µm. (b) Relative expression levels of *C. albicans* filamentous-related genes in DMSO- or mutanocyclin-treated (32 µg/ml) cells. the expression level in the DMSO control was set to 1. Error bars denote the standard deviation (SD). *P < .05 (Student's *t* test, two tailed). (c) Growth curves of *C. albicans* cells treated with DMSO (control) containing 8 µg/ml, 16 µg/ml, and 32 µg/ml of mutanocyclin. the *X*-axis and *Y*-axis represent the culture time and corresponding cell density (OD600). Bars indicate standard deviations.

To verify the inhibitory effect of mutanocyclin on *C. albicans* filamentation, we examined the dosage effect of chemically synthesized mutanocyclin. As shown in Figure 3a, mutanocyclin at concentrations of 8 µg/mL and 16 µg/mL remarkably reduced filamentation of *C. albicans* in BHI-sucrose medium after 24 hours or 36 hours of incubation, whereas it completely abolished *C. albicans* filamentous growth at 32 µg/mL. We then analyzed the expression levels of four filamentous-specific genes, *HWP1*, *ECE1*, *FLO8*, and *TEC1* [4]. In the presence of mutanocyclin, the expression levels of these genes were all notably downregulated, consistent with the morphological changes in *C. albicans* (Figure 3b).

Since the inhibitory activity of mutanocyclin on morphological changes could be due to an indirect effect, we next examined whether this chemical affected the cell growth of *C. albicans*. As shown in Figure 3c, mutanocyclin had no significant effect on the cell growth rate of *C. albicans* at different concentrations (from 8 to 32 µg/mL). Moreover, although this chemical belongs to the unacylated tetramic acids, it has an acetyl side chain but does not contain a carboxylic acid terminus (Figure 2b). The pH value of mutanocyclin in water was nearly neutral and addition of this chemical to the media did not obviously affect the pH (data not shown). Taken together, mutanocyclin could function as a specific inhibitor of *C. albicans* filamentation independent of the changes in the cell growth rate and culture pH.

### *Global transcriptional profile of* C. albicans *in response to mutanocyclin*

**Figure 4.**
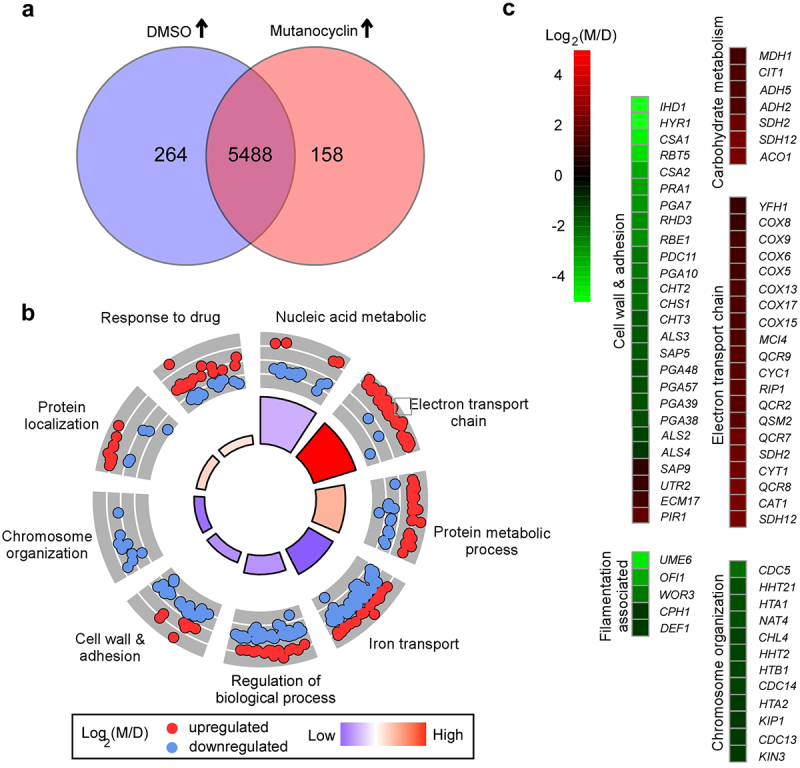
Effect of mutanocyclin on the global gene expression profile of ***C.albicans***. (a) Venn diagram depicting differentially expressed genes. A twofold difference cutoff and false discovery rate (FDRs) <.05 were used to define differentially expressed genes. (b) GO enrichment analysis of differentially expressed genes. Red or blue dots represent upregulated or downregulated genes in response to mutanocyclin treatment, respectively. The inner cycle bars represent the statistical significance. (c) Selected differentially expressed genes indicated by the R package heat map. Log2(M/D), Log2 (read counts of mutanocyclin treatment/read counts of DMSO control).

To explore the potential mechanism of mutanocyclin-regulated filamentation in *C. albicans*, we performed RNA-Seq analysis. Total RNA was extracted from cells grown in liquid Lee’s glucose medium containing DMSO or 32 µg/mL mutanocyclin for 24 hours at 30°C As demonstrated in Figure 4 and **Dataset S1**, we found 422 differentially expressed genes between the DMSO and mutanocyclin-treated samples (two-fold difference cutoff). Among them, the expression levels of 158 genes were increased and 264 genes decreased in the mutanocyclin-treated cells. The downregulated genes included a large subset of cell wall- and adherence-associated genes, for example, the GPI-anchored protein-encoding genes *IHD1*, *HYR1*, *RBT5*, and *RHD3*, cell wall surface antigen-encoding gene *CSA1*, Pry family protein-encoding gene *RBE1*, *ALS* family genes (encoding adhesins), and chitin remodeling and synthesis-associated genes *CHT2*, *CHT3*, and *CHS1*. The expression of these genes is affected by Efg1, a downstream target of the cAMP/PKA signaling pathway [39]. Some cell wall biogenesis- and remodeling-related genes, including *PIR1* (encoding 1,3-β-glucan-linked cell wall protein), *ECM17* (encoding sulfite reductase), and *UTR2* (encoding GPI-anchored cell wall glycosidase), were upregulated in response to mutanocyclin treatment. These findings indicate that mutanocyclin treatment has a strong effect on the cell wall of *C. albicans*. Interestingly, many oxidative metabolism-associated genes, including key members of the tricarboxylic acid (TCA) cycle (*CIT1*, *ACO1*, *SDH2*, *SDH12*, and *MDH1*) and electron transport chain (*COX5, COX6*, *COX8*, *COX9*, *COX13*, *COX15*, and *COX17*), were robustly upregulated in response to treatment with mutanocyclin. Numerous transcriptional regulator-encoding genes that are involved in filamentous growth or adherence in *C. albicans* were markedly downregulated in response to mutanocyclin treatment, for example, *UME6*, *OFI1*, *WOR3*, *CPH1*, and *DEF1*. This finding is consistent with the reduced filamentation ability. Moreover, a number of genes involved in chromosome organization or segregation (for example, *HHT21*, *HTA1*, *HHT2*, *HTB1*, *CDC14*, *HTA2*, and *KIP1*) were also downregulated by mutanocylin. Taken together, these results imply that mutanocylin may have a global impact on the transcriptional profile of *C. albicans*.

### *Role of the GPI-anchored proteins Spr1, Hyr4, and Iff8 in mutanocyclin-regulated filamentation in* C. albicans

**Figure 5.**
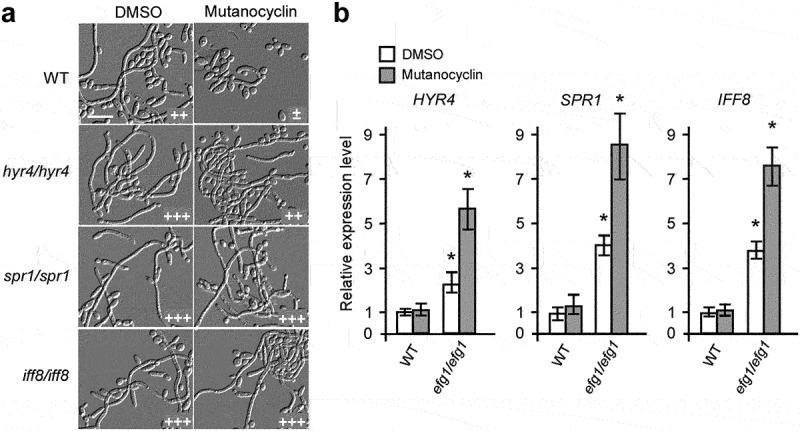
**Three cell wall-related genes are identified as negative regulators in the presence of mutanocyclin**. (a) Cellular morphologies of the *hyr4/hyr4*, *spr1/spr1*, and *iff8/iff8* mutants in the presence of DMSO or mutanocyclin. *C.* *albicans* cells (1 × 10^4^) were grown in Lee’s glucose medium containing DMSO or 32 µg/mL mutanocyclin, and incubated at 30°C for 24 hours. “±”, “++”, and “+++” represent 1–10%, 30–50%, and 50–70% of filamentous cells, respectively. Scale bar, 20 µm. (b) Relative expression levels of *HYR4*, *SPR1*, and *IFF8* in *efg1/efg1*mutants treated with DMSO or mutanocyclin (32 µg/mL). The expression level of the WT strain treated with DMSO was set as 1. Error bars denote the standard deviation (SD). *P < .05 (Student's *t* test, two tailed).

Since a set of cell wall-associated genes exhibited a differential expression pattern when treated with mutanocyclin, we examined the morphological phenotypes of a subset of cell wall-related gene mutants [40]. We screened three genes (*SPR1*, *HYR4*, and *IFF8*) that could potentially be involved in regulating the mutanocyclin response. *SPR1*, *HYR4*, and *IFF8* encode GPI-anchored proteins that are required for cell wall biogenesis and remodeling. *SPR1* (SPorulation Regulated) encodes a putative glucan 1,3-β-glucosidase that has been identified to participate in cell wall modification [41]. *HYR4* (HYphally Regulated) and *IFF8* (IPF family file) encode two proteins that belong to the Hyr/Iff cell wall family and play a role in cell wall integrity, cell adhesion, and biofilm formation [42,43]. Unlike the WT control, deletion of *SPR1*, *HYR4*, and *IFF8* allowed obvious filamentous growth in the presence of mutanocyclin (Figure 5a), suggesting that these cell wall proteins play a critical role in mutanocyclin-regulated filamentation in *C. albicans*.

Since the Efg1 transcription factor plays a critical role in the regulation of a number of cell wall-associated genes [44], we detected the expression of *SPR1*, *HYR4*, and *IFF8* in the *efg1/efg1* mutant. As shown in Figure 5b, the expression levels of all three genes were increased in the *efg1/efg1* mutant compared with the WT strain, especially when treated with mutanocyclin.

### Role of the Ras1-cAMP/PKA signaling pathway of C. albicans in the mutanocyclin-induced response

**Figure 6.**
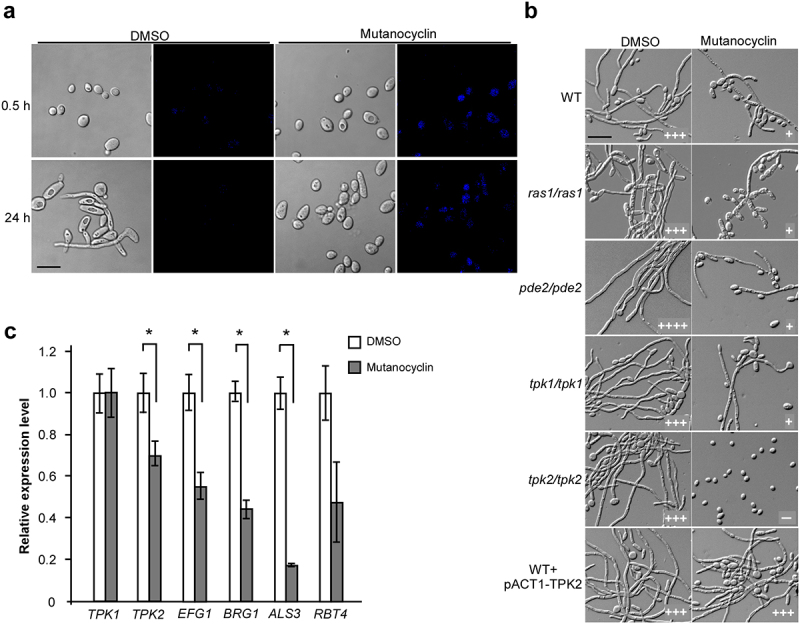
Role of the Ras1-cAMP/PKA signaling pathway in mutanocyclin-regulated filamentation in ***C.albicans***. (a) Confocal laser scanning microscopic images of *C.* *albicans* (SC5314) cells treated with mutanocyclin (blue) for 0.5 or 24 hours. Scale bar, 10 µm. (b) Cellular morphologies of the Ras1-cAMP/PKA signaling mutants and TPK2-overexpressing strain in the presence of DMSO or mutanocyclin. Fungal cells were grown in liquid Lee’s glucose medium containing mutanocyclin (32 µg/mL) or DMSO, and incubated at 30°C for 24 hours. The “-” sign indicates that no filamentous cells were observed; “+”, “+++”, and “++++”, represent 10-30%, 50-70%, and >70% of filamentous cells, respectively. Scale bar, 20 µm. (c) Relative expression levels of *TPK1*, *TPK2*, and four transcription factor-encoding genes in *C.* *albicans* cells treated with DMSO or mutanocyclin (32 µg/mL). The expression level in DMSO was set as 1. Error bars denote the standard deviation (SD). *P < .05 (Student's *t* test, two tailed).

Mutanocyclin is a tetramic acid compound that can be directly observed in fungal cells using a confocal laser scanning microscope (under UV light at 254 nm). As demonstrated in Figure 6a, mutanocyclin accumulation was observed in the cytoplasm of *C. albicans* cells, whereas no fluorescent signal was detected in the untreated control. Since the conserved Ras1-cAMP/PKA signaling pathway plays a central role in the regulation of filamentation in *C. albicans* [21,22], we tested whether mutanocyclin targeted this intracellular pathway. We found that deletion of *RAS1*, *TPK1*, and *PDE2* had no significant effect on the growth of filaments in *C. albicans*, whereas deletion of *TPK2* completely abolished filamentous development in the presence of mutanocyclin (Figure 6b). To confirm the importance of Tpk2 in the mutanocyclin-induced response, we detected overexpressed *TPK2* (strain WT + pACT1-TPK2) in *C. albicans*. As expected, the *TPK2-*overexpressing strain overrode the inhibitory effect of mutanocyclin on *C. albicans* filamentation. Consistently, q-RT-PCR analysis revealed that the expression levels of *TPK2* and its downstream targets *EFG1* and *BRG1*, *ALS3*, and *RBT4* were downregulated following mutanocyclin treatment. However, no obvious changes were detected in the expression of *TPK1* (Figure 6c). Moreover, the expression levels of the cell wall-associated genes *HYR4*, *SPR1*, and *IFF8* were significantly increased in the *efg1/efg1* mutant, which is a target of Tpk2 [45]. Therefore, the PKA catalytic subunit Tpk2, but not Tpk1, plays a crucial role in mutanocyclin-repressed filamentation in *C. albicans*.

### *Roles of transcriptional and cell wall regulators in mutanocyclin-repressed filamentation in* C. albicans

**Figure 7.**
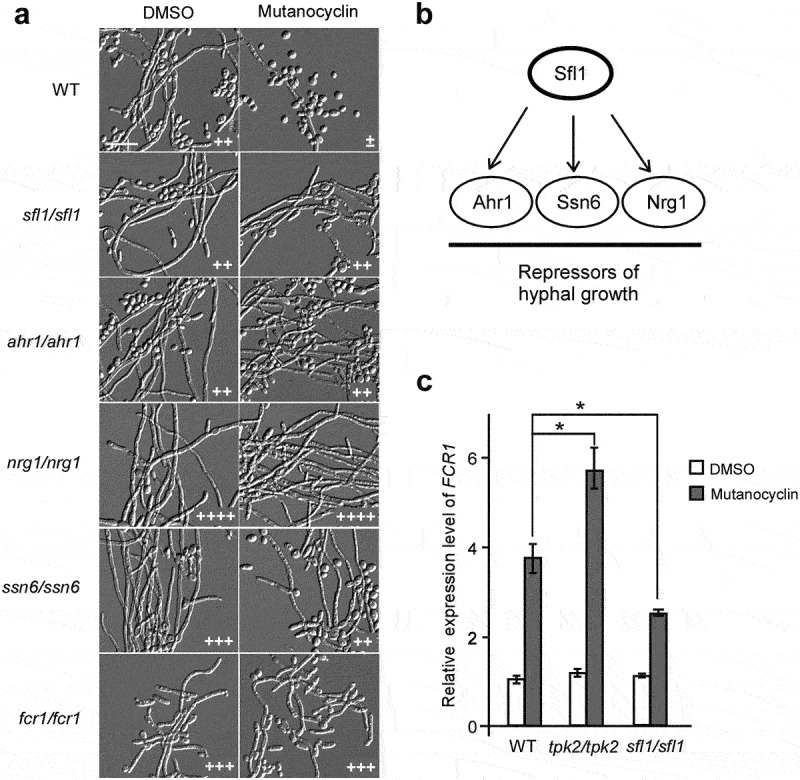
**A library screen identifies a set of transcriptional regulators involved in the response to mutanocyclin treatment**. (a) Cellular morphologies of the *sfl1/sfl1*, *ahr1/ahr1*, *nrg1/nrg1*, *ssn6/ssn6*, *fcr1/fcr1* mutants in the presence of DMSO or mutanocyclin. *C.* *albicans* cells (1 × 104) were grown inLee’s glucose medium containing DMSO or 32 µg/mL mutanocyclin, and incubated at 30°C for 24 hours. “±”, “++”, “+++”, and “++++” represent 1–10%, 30–50%, 50–70%, and >70% of filamentous cells, respectively. Scale bar, 20 µm. (b) Model of Sfl1-mediated regulation of filamentation in *C.* *albicans*. (c) Relative expression levels of *FCR1* in the WT (SC5314), *tpk2/tpk2*, and *sfl1/sfl1* strains treated with DMSO or mutanocyclin (32 µg/mL). The expression level of the WT strain treated with DMSO was set as 1. Error bars denote the standard deviation (SD). *P < .05 (Student's *t* test, two tailed).

To further explore the molecular mechanism of mutanocyclin, we screened a deletion mutant library of 165 transcription factors [32]. The filamentation assays were performed in Lee’s glucose medium containing 32 µg/mL mutanocyclin or DMSO (control). Five mutants (*sfl1/sfl1*, *ahr1/ahr1*, *ssn6/ssn66*, *nrg1/nrg1*, and *fcr1/fcr1*) were able to undergo filamentation even in the presence of mutanocyclin (Figure 7 a), suggesting that these genes played a negative role in filamentous growth under these culture conditions. It has been reported that Sfl1 (suppressor gene for flocculation 1) is a specific target of Tpk2 and acts as a negative regulator of filamentation in *C. albicans* [46]. This result is consistent with findings obtained for the *tpk2/pk2* mutant and TPK2-overexpressing strain (Figure 6 b). Ahr1, Nrg1, and Ssn6 are also well-characterized regulators of filamentation, which have been demonstrated to be regulated by Sfl1 in *C. albicans* [46] (Figure 7 b).

Fcr1 is a predicted zinc cluster transcription factor that functions as a negative regulator of fluconazole and ketoconazole resistance, as well as in the regulation of filament formation [47]. We found that deletion of *FCR1* allowed obvious filamentous growth in the presence of mutanocyclin. Consistently, expression of the *FCR1* gene was notably enhanced by mutanocyclin treatment (Figure 7c), suggesting that Fcr1 is a key regulator in the response to mutanocyclin. Znaidi *et al*. reported that Fcr1 is a transcriptional target of Sfl1 in various biological processes [46]. Q-RT-PCR assays demonstrated that compared with the WT strain, the expression level of *FCR1* was increased in the *tpk2/tpk2* mutant and decreased in the *sfl1/sfl1* mutant following treatment with mutanocyclin (Figure 7c). This expression pattern is consistent with the morphological phenotypes of these mutants in the presence of mutanocyclin.

### *Efficacy of mutanocyclin in ex vivo mouse tongue and* G. mellonella *infection models of* C. albicans

**Figure 8.**
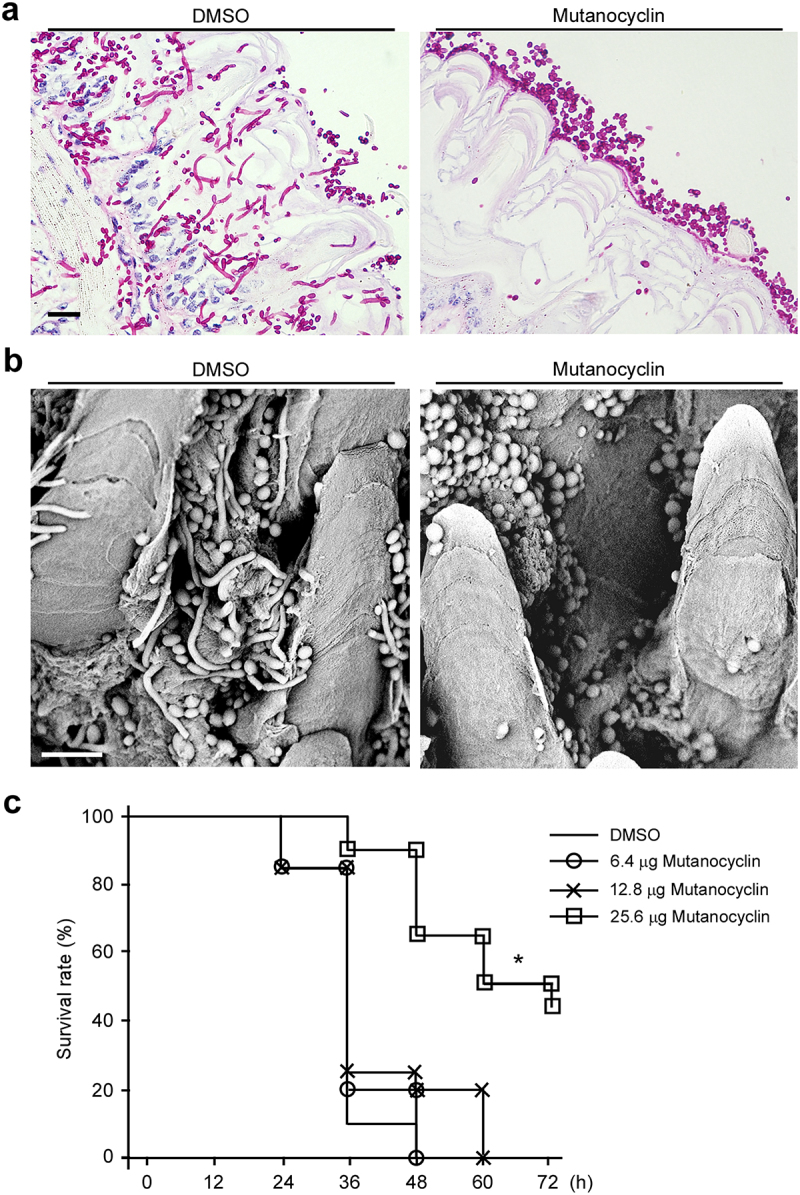
Effects of mutanocyclin on the virulence of ***C.albicans*** in mouse and ***G. mellonella*** infection models. (a) Histopathological assays. *C.* *albicans* SC5314 cells (1 × 10^7^) in 5 μL of PBS containing DMSO or mutanocyclin (32 μg/mL) were spotted on the removed tongues of 4 − 5-week-old mice. After 24 hours of infection at 37°C, the infected tongues were stained with PAS and then used for microscopy assays. Scale bar, 20 µm. (b) Scanning electron microscope (SEM) images of the infected tongue tissues. Scale bar, 10 µm. (c) Survival rates of *C.* *albicans*-infected *G.* *mellonella* larvae treated with DMSO or mutanocyclin. *C.* *albicans* SC5314 cells (1 × 10^8^ CFU/mL) and DMSO, or 6.4, 12.8, or 25.6 µg mutanocyclin, were injected individually into each *G.* *mellonella* larva (20 larvae in each treatment group). *P < .05 (compared with the DMSO control, Student's *t* test, two tailed).

Filamentous growth is important for the virulence of *C. albicans*. Given the potent inhibitory effect of mutanocyclin on filamentous growth of *C. albicans*, we predicted that treatment with this chemical would reduce fungal virulence. First, we performed ex vivo mouse tongue infection assays. As shown in Figure 8a, b, *C. albicans* cells in the DMSO-treated control formed robust filaments and invaded the tongue tissue. However, *C. albicans* cells in the mutanocyclin-treated sample maintained the yeast-form. These results imply that mutanocyclin can function as a filamentation repressor during infection and has potential as a therapeutic agent.

We next performed in vivo virulence assays using the *G. mellonella* infection model to evaluate the efficacy of mutanocyclin. *G. mellonella* larvae were infected with *C. albicans*, and the survival rates were analyzed. As shown in Figure 8c, the groups infected by *C. albicans* and treated with DMSO or 6.4 µg mutanocyclin showed 100% of death within 48 hours postinfection. However, the survival rate was increased by 20% and 60% following treatment with 12.8 µg and 25.6 µg of mutanocyclin, respectively. These results suggest that mutanocyclin notably attenuates the virulence of *C. albicans* in the *G. mellonella* infection model.

## Discussion

Cross-kingdom interactions between bacteria and fungi are common and important for infection outcomes. The symbiotic relationship between *S. mutans* and *C. albicans* has been well investigated [11,13,14,48]. For example, *C. albicans* secretes the quorum-sensing molecule, farnesol, to stimulate microcolony and biofilm development of *S. mutans* [11]. Animals coinfected with the two species display higher levels of microbial carriage and lesion severity [13]. In this study, we report a novel antagonistic relationship between the two species: *S. mutans* suppresses the filamentous growth and virulence of *C. albicans* through secreting a secondary metabolite, mutanocyclin. This chemical has a tetramic acid reutericyclin core in its structure [34]. We demonstrated that mutanocyclin regulated morphological transitions in *C. albicans* through multiple mechanisms including alterations of the Ras1-cAMP/PKA pathway, transcriptional regulation, and cell wall biogenesis/remodeling. These changes finally resulted in its therapeutic effect on *C. albicans* infections in mouse skin and in *G. mellonella* infection models.

The Ras1-cAMP/PKA signaling pathway plays a critical role in filamentous growth of *C. albicans* [21,22]. Here, we found that the catalytic subunit of PKA, Tpk2, functioned as a major regulator in mutanocyclin-repressed filamentous growth. Inactivation of Tpk2 led to complete abrogation of filamentous growth in the presence of mutanocyclin, whereas overexpression of *TPK2* was able to override the suppressive effect of this chemical (Figure 6b). Consistently, expression of the downstream target gene *EFG1* and cell wall-associated genes *ALS3* and *RBT4* were significantly decreased following treatment with mutanocyclin. Moreover, dysfunction of *HYR4, SPR1*, and *IFF8*, which encode three GPI-anchored proteins associated with cell wall biogenesis and remodeling, allowed obvious filamentous growth in the presence of mutanocyclin. Deletion of *EFG1* led to increased expression of *HYR4*, *SPR1*, and *IFF8* in *C. albicans*, especially when treated with mutanocyclin (Figure 5). These results suggest that both transcriptional regulation and cell wall biogenesis are involved in the regulation of mutanocyclin-inhibited filamentous growth of *C. albicans*.

The Sfl1 transcription factor is a negative regulator of filamentous growth that physically interacts with Tpk2 [46]. In *Saccharomyces cerevisiae*, Tpk2 acts upstream of Sfl1 in the regulation of pseudohyphal growth through phosphorylation of Sfl1. This phosphorylation relieves the repression of genes (for example, *FLO11*) regulated by Sfl1 [49]. In *C. albicans*, Tpk2 also bound preferentially to Sfl1, and deletion of *SFL1* allowed the development of filaments even in the presence of mutanocyclin (Figure 7), suggesting the presence of a conserved regulatory mechanism in the two yeast species. The *ahr1/ahr1*, *ssn6/ssn6*, and *nrg1/nrg1* mutants were able to undergo filamentation in the presence of mutanocyclin (Figure 7). These regulators and Sfl1 comprise a network in which Sfl1 acts as a central “switch on/off” regulator that plays a crucial role in filamentous growth of *C. albicans* in response to mutanocyclin (Figure 7).

Transcription of the zinc cluster regulator Fcr1 was enhanced in *C. albicans* cells following treatment with mutanocyclin (Figure 7). It has been reported that Fcr1 contains a PKA phosphorylation site and an ATP/GTP binding motif, suggesting that Fcr1 is a potential target of Tpk2 [47,50]. Disruption of *TPK2* causes a significant increase in *FCR1* expression. Moreover, Sfl1 has also been demonstrated to bind to the promoter of *FCR1* [47], and deletion of *SFL1* leads to reduced expression of *FCR1* following treatment with mutanocyclin. These results suggest that Tpk2 and Sfl1 play a major role in the response to mutanocyclin treatment and that *FCR1* is a downstream factor of the two regulators.

**Figure 9.**
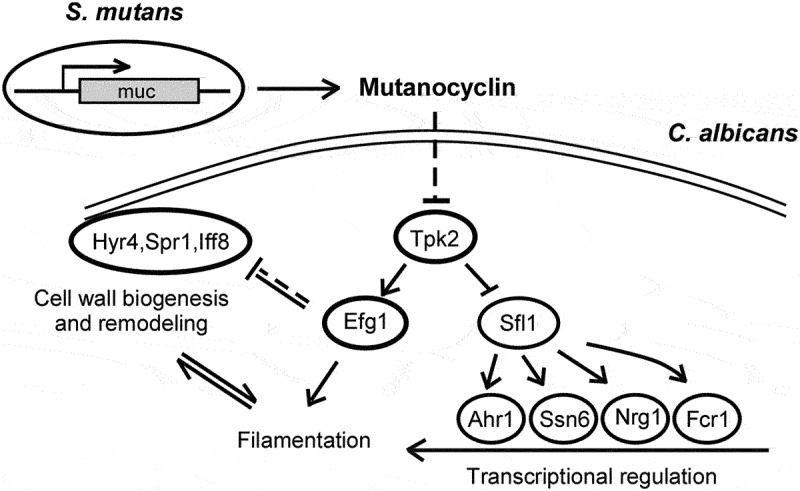
Model for the mutanocyclin-mediated interspecies interaction between *C.* *albicans* and *S.* *mutans*. *S.* *mutans* secretes the secondary metabolite, mutanocyclin, which regulates filamentation of *C.* *albicans* through the Ras1-cAMP/PKA signaling pathway and a set of transcription factors and cell wall-associated proteins.

RNA-Seq analysis demonstrated that mutanocyclin treatment had a global effect on the transcriptional profile in *C. albicans* (Figure 4, Dataset S1). A large set of genes involved in metabolism, cell wall remodeling, and chromosome organization were differentially expressed in response to treatment with this chemical. These changes could directly or indirectly affect filamentous or polarized growth in *C. albicans*. The Ras1-cAMP/PKA signaling pathway and Efg1 have also been demonstrated to function in the regulation of cell wall biogenesis [51–53]. Therefore, the transcriptional profile reflects the outcome of the regulation of corresponding signaling pathways following treatment with mutanocyclin. This chemical could modulate cell wall components predominantly through the Ras1-cAMP/PKA signaling pathway and Efg1, as well as a subset of filamentous regulators through Sfl1. We propose that this transcriptional regulation and cell wall alterations coordinately regulate the response to mutanocyclin in *C. albicans* (Figure 9).

Mutanocyclin did not affect the cell growth rate of *C. albicans* even at a high concentration, but it specifically targeted filamentous growth. The development of filaments is critical for *C. albicans* to cause infections. It has been demonstrated that *C. albicans* filaments are produced coordinately with virulence factors such as adhesins and hydrolytic enzymes andgeneration of hydrostatic pressure [54,55]. *C. albicans* filaments can actively penetrate and rupture the macrophages, thereby conferring the pathogen cells greater resistance to the host immune system [56]. Thus, targeting the regulatory process of filamentous growth could be a promising strategy for anti-*C. albicans* infection treatments. Furthermore, our virulence assays in two animal models revealed a notable therapeutic effect of mutanocyclin. Fcr1, a potential target of mutanocyclin, has been described as a suppressor of fluconazole and ketoconazole resistance [47], implying a synergistic effect between mutanocyclin and azole drugs. Taken together, we report a novel cross-kingdom relationship between *S. mutans* and *C. albicans*. Since both species are commensal microorganisms of the mouth niche, the inhibitory effect of *S. mutans* on filamentous growth of *C. albicans* could be critical for the biofilm dispersal process. Additionally, because the increasing prevalence of drug-resistant pathogenic fungi threatens the effectiveness of existing antifungal agents, we propose that mutanocyclin, or similar compounds, may provide a novel therapeutic potential for future applications.

## Data Availability

All primary data that support the findings of this study are available from the corresponding author upon reasonable request.

## Accession number

The RNA-seq dataset was deposited in the NCBI Gene Expression Omnibus (GEO) portal with accession number GSE188886.
